# Measuring sustainability of opioid agonist therapy programs in the context of transition from Global Fund support

**DOI:** 10.1186/s12954-024-00931-0

**Published:** 2024-01-12

**Authors:** Raminta Stuikyte, Ivan Varentsov, Catherine Cook, Sergii Dvoriak

**Affiliations:** 1 Vilnius, Lithuania; 2 Eurasian Harm Reduction Association (EHRA), Vilnius, Lithuania; 3 Harm Reduction International, London, UK; 4 Ukrainian Institute on Public Health Policy, Kyiv, Ukraine

**Keywords:** Opioid agonist therapy, Transition, Sustainability, Funding, Injecting drug use, Eastern Europe, Central Asia

## Abstract

**Background:**

Programmatic and financial sustainability of health responses dependent on donor funding has risen as a major concern. In the HIV field in particular, it generated a number of instruments and assessments on sustainability and processes related to donor transition planning. The authors aimed to develop an instrument specific to opioid agonist therapy (OAT) programs as they were addressed only marginally by the HIV-specific assessments.

**Methods:**

The development of the OAT sustainability instrument used desk review of existing HIV sustainability concepts and tools, an International Advisory Board, and piloting to validate the instrument.

**Results:**

The new OAT sustainability instrument is comprised of the three parts: the conceptual framework, methodological guidelines and a practical implementation tool for assessing the degree of OAT sustainability at the country level. It measures sustainability in the three broad areas for sustainability measuring–Policy & Governance; Finance & Resources; and Services. The selection of indicators and their composites for the three sustainability areas extensively used the United Nations and World Health Organization’s guidance on health system building blocks, on care and HIV and viral hepatitis prevention among people using opioids and for opioid dependence, and the definition of access to health framed by the United Nations Convent on Economic, Social and Cultural Rights. The instrument’s methodological guidelines require the engagement of a national consultant to conduct desk review, key informant interviews and focus groups for measuring discrete milestones and adding qualitative information for interpretation of the data, progress and opportunities. The guidelines advise engaging a country-specific multi-stakeholder advisory group for planning, validation and follow-up of the assessment. The pilot of the instrument in 3 countries in 2020 validated it and required minor adjustments in the instrument. By mid-2023, the instrument has been successfully applied in 5 countries.

**Conclusions:**

The developed instrument enables a comprehensive review of the resilience of OAT programs and their ability to scale up and to inform a roadmap for improved sustainability. While developed in the context of Eastern Europe and Central Asia, it has been reviewed by a global advisory panel and could be easily adapted outside this regional context.

## Background

In the last decade, the sustainability of health responses that are dependent on donor funding has risen as a major concern, especially in the HIV field [1, 2]. There is no one internationally agreed definition for sustainability of health programs that would work for all health issues including non-communicable health concerns. The Global Fund to Fight HIV/AIDS, Tuberculosis and Malaria defines sustainability as the “ability of a health program or country to both maintain and scale up service coverage to a level, in line with epidemiological context, that will provide for continuing control of a public health problem and support efforts for elimination of the three diseases, even after the removal of external funding by the Global Fund and other major external donors” [3]. Increasing and ensuring the continued reliability of financing is an important component of sustainability. Other key components relate to programmatic aspects, including leadership and governance, overcoming structural barriers (such as those related to human rights), increasing efficiencies, improving integration in health systems and ensuring epidemiological control through high, impactful coverage of service delivery. In 2016, the largest international harm reduction donor, the Global Fund to Fight HIV/AIDS, Tuberculosis and Malaria (in short, the Global Fund) [4], adopted the Sustainability, Transition and Co-Financing Policy and dedicated one of the four pillars of its strategy for 2017–2022 to building resilient and sustainable systems for health [3, 5]. The Global Fund set differentiated expectations for countries falling at different places of the sustainability and transition continuum, based on the income status and the epidemic levels, with higher expectations of self-reliance and co-financing from domestic public resources from upper-middle-income countries, closer to transitioning out from the donor’s support. Several HIV frameworks and practical tools for sustainability and transition have been developed [6] and applied in countries classified by the World Bank as upper- and lower-middle income.

Opioid agonist maintenance treatment or opioid agonist therapy (OAT),^1^ combined with psychosocial assistance, is the most effective modality of managing opioid dependence, recommended by the World Health Organization (WHO) [7]. It is an essential component of HIV prevention, treatment and care programs among people who use opioids [8]. Still, the HIV frameworks and tools on sustainability and donor transition have marginal focus on OAT sustainability. They are limited to mentions of financial handovers of OAT from donor to domestic sources and the scale of services, without further analysis [9].

The Eurasian Harm Reduction Network developed a transition readiness assessment tool for harm reduction which included OAT [10]. However, in practice this tool’s framework and application focused on the components of harm reduction delivered by non-governmental organizations without analysis and recommendations for systemic building of OAT sustainability which, in Eastern Europe and Central Asia (EECA) and in many countries globally, is under the remit of the public healthcare system.

Domestic funding for OAT programs lags far behind the needs. In last count, harm reduction funding in low- and middle-income countries totaled US$131 million—just 5% of the resources UNAIDS estimates to be required annually by 2025. Additionally, just half of it came from domestic sources, remaining half continuing to depend on the Global Fund and other international support [11]. In the EECA region, the introduction and often the development of OAT and broader harm reduction was implemented part of the response to HIV [12, 13]. Therefore, traditionally it has been funded from the HIV budgets, not by the state drug treatment system. Moreover, in EECA, a significant factor limiting domestic financing and political support for OAT is stigma inherited from the Soviet Union [14, 15]. Stigma related to drug use, people who use drugs and the use of opioids in medicine remains high in the society including among health professionals [16–18]. Consequently, public resource allocations have often been dependent on residual budgets in HIV and drug treatment and are a recurrent opportunistic target for political parties ahead of elections [14]. In this donor transition period, an objective analysis of the progress and gaps, supportive factors and opportunities are crucial to informing political, technical and financial dialogue to move towards sustainable OAT programs.

## Methods

An instrument was constructed to guide objective in-country assessment for measuring progress and risks to programmatic and financial sustainability for OAT programs in countries transiting from the Global Fund and other international donor support.

The Eurasian Harm Reduction Association (EHRA) commissioned and facilitated the work as part of their continued work to support sustainable harm reduction programming in the EECA region. Additionally, an International Advisory Group was set up to support the process, specifically to agree on the conceptual principles of the framework, review methodology, prioritize indicators and align terminology. The 9-member International Advisory Group included diverse perspectives of OAT practitioners, technical support providers, World Health Organization (WHO), technical experts from the two donors of harm reduction (Open Society Foundations and the Global Fund to Fight HIV/AIDS, Tuberculosis and Malaria), and two global networks—International Network of People Who Use Drugs and Harm Reduction International.

To inform the design of the instrument, in 2019–2020 the authors conducted a desk review of existing sustainability frameworks, instruments and lessons from their application in the global health sector, adapted the sustainability measuring concepts, designed a specific instrument for OAT and piloted it for its acceptability and feasibility before its finalization.

As the first step, the desk review prioritized the following three sources for defining the methodological approach: Transition Preparedness Assessment Framework developed by Curatio International Foundation [19], Transition Readiness Assessment Tool [10] by Eurasian Harm Reduction Network and an Oberth and Whiteside’s expanded understanding of sustainability that encompasses a human rights component [2]. The International Advisory Group helped prioritize and confirm the dimensions, the indicators and the potential benchmarks.

Following analysis of existing approaches to measuring sustainability, a conceptual framework was established to understand and measure OAT sustainability at the country level. Furthermore, the additional review of the normative guidance on transition and opioid agonist therapy identified a set of composites of indicators and benchmarks, which were further prioritized by the International Advisory Group. The conceptual framework informed the design of a practical tool for country-level assessment and accompanying practical methodology guidelines. Hence, the new instrument is comprised of the conceptual framework, the methodological guidelines and the practical tool.

The EHRA, in consultation with UNAIDS, identified two countries for piloting the implementation of the tool, selecting diverse settings: one country classified as a lower-middle-income country by the World Bank in Central Asia (Tajikistan) and another Eastern European upper-middle-income country (Belarus). Previously, civil society from both countries expressed their concerns over OAT’s sustainability. Additionally, one donor representative from Ukraine suggested funding a pilot in Ukraine, which was undergoing significant health reforms and had the largest investment from the Global Fund due to its large size and significant HIV epidemic. The national consultants submitted their structured feedback on the instrument and its implementation process as part of the piloting outputs. This feedback guided the final revisions of the instrument.

## Results

Based on lessons learned from the previous sustainability assessments, the authors aimed to adhere to the following four principles in the design and methodology of the OAT instrument:

1. Increase national stakeholder ownership of the results of the national assessments;
2. Improve feasibility to settings with limited capacity and funding;
3. Minimize time required for country assessment, while retaining a systemic approach, to facilitate timely recommendations for action; and
4. Assure objectivity in the assessment.

### Conceptual framework

The OAT sustainability framework breaks down the concept of sustainability into a matrix of key indicators, grouped into the following three broad dimensions required for continuation and scale-up of OAT: Policy & Governance; Finance & Resources, and Services. The dimension of Policy & Governance not only addresses the issues of political commitment and supportive legislation, legal regulation and governance for OAT but also includes indicators on the management of smooth donor transition. Indicators under Finance & Resources are the critical inputs of health systems for OAT, based on the WHO’s concept of health system blocks [20]. The last dimension on Services measures the level of access to OAT, adapting the concept of the critical elements of the right to health suggested by the UN Committee on Economic, Social and Cultural Rights^2^: (1) availability; (2) accessibility (non-discrimination, physical accessibility, economic accessibility or affordability, information accessibility); and (3) quality and integration. Acceptability is not included as this more nuanced aspect requires a representative assessment among OAT clients which is outside the scope of the instrument. The priority indicators and their corresponding composites extensively rely on global normative guidance. WHO/UN guidance on opioid dependence management, responses to HIV, viral hepatitis among people who use drugs and the previous reviews on the measuring of the quality of OAT [21–26] informed the design of the OAT sustainability instrument, especially in the dimension of Services. An indicator on donor transition management was added based on lessons from transition assessments developed in EECA, i.e., that the transition needs to be planned, its implementation governed and relevant measured funded.

### Measurement tool

#### Dimensions and indicators

The conceptual sustainability framework identified three dimensions, each with 2–4 corresponding indicators. In total, there are nine indicators across these dimensions, as indicated in Table 1. Each dimension is considered equal, though the number of indicators varies.

**Table 1:** Dimensions and indicators

Dimensions	Indicators					
A. Policy & Governance	A1. Political commitment			A2. Management of transition from donor to domestic funding		
B. Finance & Resources	B1. Medications	B2. Financial resources		B3. Human resources		B4. Evidence and information systems
C. Services	C1. Availability and coverage		C2. Accessibility		C3. Quality and integration	

#### Composites of indicators and measurement scales

**Fig. 1:**
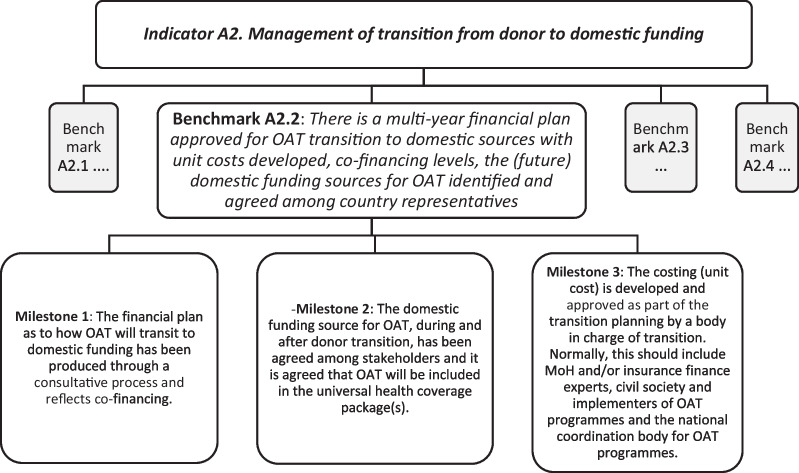
Illustration of the granular approach taken to measure progress on indicators through benchmarks and milestones

Performance on each indicator is measured through a set of 4–6 benchmarks, which are broken down into a set of milestones, where relevant referencing specific normative guidance. Each milestone is framed as a discrete statement seeking to reduce subjectivity to the degree possible. Figure 1 provides an example of the granular measurement approach taken for one indicator.

**Fig. 2:**
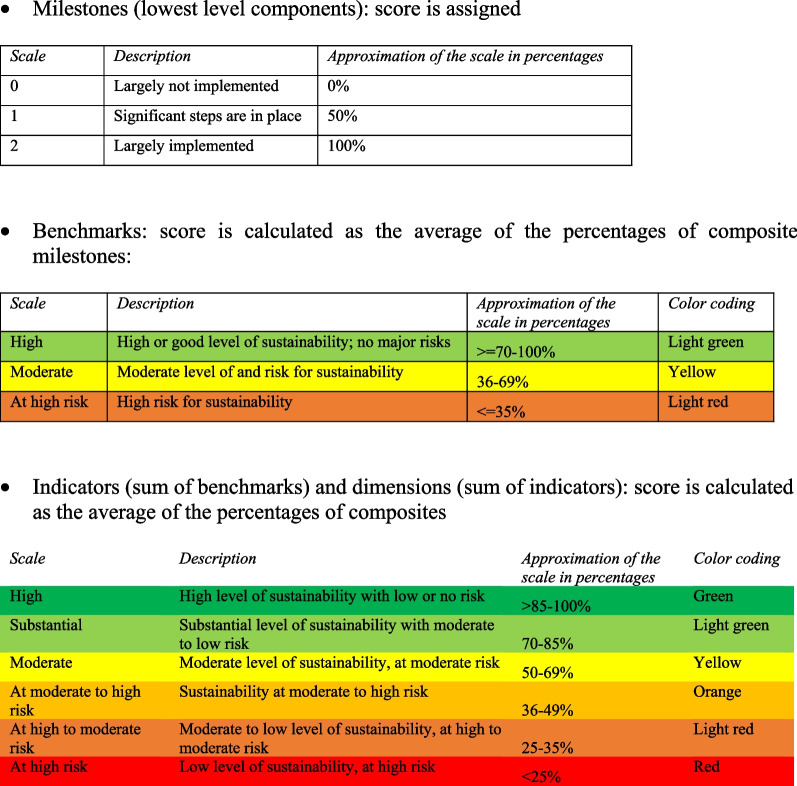
Scales and scoring for calculating status of sustainability

Implementation of the instrument allows researchers to establish the extent to which milestones have been met in a specific setting. Each milestone is scored using the three-level scale. The measurements of the remaining higher-level components of the framework—benchmarks, indicators and dimensions—are automatically calculated as average composites. In alignment with existing sustainability frameworks [19], the instrument uses a three-level sustainability scale for benchmarks and a six-degree scale for indicators and dimensions, ranging from high degree of sustainability to sustainability being at high risk, as indicated in Fig. 2.

The instrument contains additional templates to facilitate the collection of core markers for data-driven analysis of several benchmarks related to funding, human resources, service accessibility, integration and quality, among others. The data for these markers demonstrate trends over years and/or present details on benchmarks. For example, the funding landscape template covers the last 5 years and map funding sources for the procurement of medicines, human resources and other different inputs required for OAT planning, monitoring, service delivery and quality assurance.

#### Additional data collected for qualitative analysis

In addition to measuring each milestone and backing it with data sources, the instrument records qualitative data to interpret the milestone scoring and at each benchmark level to add important context to scoring including the trends over the last 3–5 years, recorded factors for progress and challenges, aspects demonstrating how this benchmark is impacted by transition, and identify upcoming government processes and other opportunities for improved sustainability.

### Methodological guidelines

The implementation of the instrument requires a national consultant who is knowledgeable of OAT in the country and has access to the national documentation and stakeholders but remains significantly independent (i.e., the national program manager of OAT could not serve as the consultant due to a conflict of interest for the implementation of the tool).

The guidelines advise engaging a country-specific multi-stakeholder advisory group for the assessment’s planning, validation and follow-up. The national consultant decides if to engage such a group based on the national context. In some contexts, securing the participation of national government officials in an advisory body requires significant time and permissions, which are challenging from a practical perspective. However, if an advisory group is not engaged, the consultant has to conduct alternative arrangements to validate the report on the tool’s implementation.

The methodological guidelines of the instrument guide a phased national assessment, once the process is set. First, desk review is steered by a list of potentially relevant documentation contained within the instrument. The guidelines guide the selection of key informants and their interviews to fill gaps and provide qualitative information. If feasible, especially in larger countries, the guidelines recommend conducting focus groups with expert OAT consumers and/or OAT practitioners. Annexes include templates for key informant interviews, focus groups and filling the instrument.

The national expert is expected to spend 10–14 work days but the total duration is planned for 2–3 months depending on the speed of accessing documents and the need for placing inquiries to government institutions or the need for getting permissions for the assessment and interviews.

### Piloting and changes in the instrument

The instrument was piloted in 2019–2020 in three countries (Belarus, Ukraine and Tajikistan). The national consultants were selected through an open call for nominations (Belarus and Tajikistan) or engagement of an International Advisory Group’s member from the country (Ukraine). In one case, it was a practitioner of OAT acting as an independent consultant, in other two cases independent consultants from civil society sector were engaged.

The pilot confirmed relevance of the majority of indicators, benchmarks and milestones independent of setting and health system model. There were three revisions as a result of the pilot. First, three benchmarks and two milestones were found to be highly dependent on the context (e.g., feasibility of relying on private providers for expansion of OAT, presence of Global Fund grant with co-financing requirements for OAT) and so the revised instrument includes these as complementary. Secondly, following the pilot, some wording had to be nuanced to reflect differences in health systems, for example, general state budget vs national health insurance used for pooling of public health financing and differentiation of lists and policies for essential and reimbursed medicines. Thirdly, all pilot countries used advisory groups, however, in practice their roles varied. In one case, it was used only for planning but not for validation of results and recommendations, due to the initiation of the COVID-19 epidemic. We did not assess the effectiveness of the advisory groups; still, using such groups remained a strong recommendation in the instrument’s methodological guidelines but not a requirement.

The practical implementation of the tool took six months, starting from the selection of the consultants until the validation and publishing of the report. Based on the feedback, the automated tool for measuring the assessment was updated and exported to the Excel-based instrument for greater automation of the calculation of scoring of the dimensions, the indicators and the benchmarks. Despite valuing the methodological guidelines, the national consultants appreciated an induction session and continuous technical support from the EHRA throughout the tool’s implementation, including during the report’s validation.

### Country assessment

Following the pilot, as of September 2023, the revised instrument has been used for re-assessment of OAT sustainability in the three pilot countries and has been applied in an additional two countries: Albania and Moldova.

The assessment and re-assessment results will be published in a separate article; therefore, they are not presented here. The sustainability assessment instrument and country reports for Albania, Belarus, Moldova, Tajikistan and Ukraine are available online at: https://eecaplatform.org/en/oat-a-guide-for-assessment-in-the-context-of-donor-transition/

## Discussion

A scoping review of the available instruments on sustainability and transition of health programs from donor to domestic funding identified no OAT or drug treatment-specific instruments [27]. The authors’ desk review corroborated this finding, yielding very little analysis of OAT sustainability, limited to a light touch on OAT within broader analysis of HIV program sustainability or general status and funding of OAT implementation programs in a specific setting. Therefore, we consider this paper and instrument to be first in the field.

OAT program development remains highly fragile and dependent on support through national HIV program budgets. The instrument considers OAT as an integral part of national drug treatment systems, unlike the existing HIV-specific tools, which consider only the HIV response. The instrument allows OAT sustainability to be assessed as part of a comprehensive health system, including within its scope but not limited to different aspects of governance, policy, access to medicines, human resources and information systems. Its design enables the practical operationalization of the use of the UN/WHO guidance on OAT in country multi-stakeholder processes. Despite a relatively systemic approach, the instrument is feasible to implement within resource and time-constrained settings, offering a low-cost national assessment requiring 6 months.

In September 2023, twelve assessment implementers, members of national advisory groups for assessments, OAT managers and OAT funders provided their anonymous feedback on the usefulness and other lessons learned from the use of the instrument in four countries. In three out of the four countries, specific examples of the use or a reference to the country assessments were reported: the National Program on HIV Prevention and Control 2021–2025 (Moldova), official presentations by the management of the state drug treatment programs to the public and government officials (Belarus and Tajikistan), the country’s funding requests to the Global Fund (Belarus and Moldova), a meeting of the Parliamentary Commission on Health (Moldova) and the NGO shadow report to Universal Period Review of Moldova at the United National Human Rights Council [28]. In Belarus, the findings were used as evidence to support a request for increased government support. Tajikistan assessment’s advisory group members see the report as an important overview of the current status (and not only the sustainability progress) for OAT. In Moldova, the assessment in 2020 led to the formation of a working group on OAT, which directly used the findings and recommendations for in-country dialogues on the national HIV program, its request to the Global Fund, etc.

Ukraine is the only country where the assessment in 2020 has not directly resulted in wider awareness or use outside some OAT experts. In part due to the COVID-19 pandemic, the assessment was highly independent from government institutions, led by an expert from a specialized analytical and research institution but did not engage the national OAT leadership and funders in planning and validation and did not use the Advisory Group to plan the follow-up, potentially resulting in limited visibility of the assessment. Additionally, the country already has a significant number of various analysis and discussions on OAT and insufficient efforts to promote the results of the report. (For example, the report has not been translated into Ukrainian and English.) This points to the importance of linking the instrument to advocacy efforts and potentially more specific advocacy funding in addition to financing of the assessment.

Despite the special attention to harmonized approaches, the country assessments using the instrument varied. In two countries, desk review identified a limited number of public data sources, and therefore, these assessments had to rely on data received through government experts with access to internal government databases and expert assessment, requiring more data analysis.

The feedback from the twelve stakeholders in September 2023 also confirms the importance of continuing to carefully balance the three principles in choosing the assessors and other steps in planning country assessments. On the one hand, a high degree of independence is important for producing an objective analysis, based on factual data and informed by but independent of the chief drug treatment doctors’ perspectives. On the other hand, the process should be consultative at the stages of planning, implementation, validation and the use of the results. Thirdly, engaging a highly motivated assessor linked to advocacy can ensure greater utilization of the results in the follow-up. The EHRA played an important role in supporting the delicate balance, as it chose and contracted the consultant in a consultative way, trying to navigate the context of power balance and expertise pool in the country. Similarly, in-country consultants have a critical role in adhering to the guidance within their country contexts. Based on the retrospective analysis of the visibility of the 2020 results and follow-up from the 2022/2023 assessments, engaging multi-stakeholder advisory groups prior to, during the assessment and/or for validation of the report among key stakeholders from government, academia, civil society and funders will be insisted for future assessments.

Stakeholder feedback highlighted the importance of regular re-assessment of OAT sustainability. However, expectations from such repeated processes might differ in countries or among stakeholder groups, therefore, require a significant consultation on the scope at the initial stage. For example, Moldova’s re-assessment narrowed its indicator *Management of transition from donor to domestic funding* to psychosocial support, which remains funded by donors and have not repeated the measurement of this indicator for other elements of OAT previously identified as well-progressed, stable in terms of self-reliance. A stakeholder from Tajikistan highlighted importance of a comprehensive approach for their country due to lack of comprehensive reports on the subject.

The sustainability measurement and result dissemination have been focused on the national level and in the future could better engage stakeholders from the sub-national level. Additionally, the instrument methodology foresees limited engagement of OAT clients as interviewees or via one focus group and utilization of assessments of OAT clients. Clients have major insights on service accessibility, availability, quality and integration. Therefore, the instrument application could be used in combination with community-led monitoring instruments on OAT, especially in relation to the indicator on the Services dimension of the instrument.

While each indicator is of equal importance within the sustainability framework, in practice indicators within the first dimension have greater weight since there are only two indicators and those in the dimension of Finance & Resources comprise four indicators and, consequently, have less weight. Interpreting assessment results therefore requires an analysis of scores under each dimension and indicator. Qualitative analysis is key to understanding the situation and identifying ways forward. For this reason, the instrument intentionally does not provide a final percentage of sustainability. However, a single sustainability score may be useful for country comparison or comparison of the country’s progress over time.

One of the limitations of the instrument is the defined list of benchmarks and milestones used. To make the instrument practical, its development involved short-listing of various aspects contributing to the resilience and access of OAT. But, as a result, the product reflects a partial picture of situation covering sustainability.

When the instrument was conceptualized, the EHRA expected that the new instrument would be taken up and promoted by WHO, the Global Fund, Harm Reduction International and other groups represented in the International Advisory Board. However, this did not happen. In part, the COVID-19 pandemic in 2020–2021 derailed attention from sustainability to the pandemic mitigation. Shifts in the Open Society Foundations’ priorities reduced resources available for civil society led research and advocacy in the field of harm reduction. Additional efforts are needed for increased uptake in the future. This should include exploring if WHO, UNAIDS, the Global Fund and similar agencies with a stake in OAT and sustainability would be willing to co-own and work on further updates and the implementation of tool. Their support would enable the greater use of the tool and the products of its implementation for advocacy.

## Conclusions

The developed instrument enables a comprehensive review of the resilience of OAT programs and their ability to scale up and to inform the development of paths forward. The assessment methodology has been effectively implemented in settings with diverse legislative and governance models and health systems, at different stages of donor transition in Eastern Europe and Central Asia. While developed in the EECA context, the three instrument’s parts—the conceptual framework, the methodological guidelines and the tool—were reviewed by a global advisory panel and could be easily adapted outside this regional context. The instrument produces outputs highlighting specific elements that could inform a roadmap for improved sustainability of OAT programs. It should be used in combination with community-led monitoring tools on service access. The collaborative approach to the assessment, with the engagement of key stakeholders responsible for OAT through an advisory group, has been proven to support greater follow-up and ownership, while might affect the ability of stronger articulation of recommendations.

## Data Availability

The instrument described in this article is available online at: https://eecaplatform.org/en/oat-a-guide-for-assessment-in-the-context-of-donor-transition/

## Appendix

See Table

**Table 2:** Sustainability framework: dimensions, indicators and benchmarks

Dimensions	Indicators and benchmarks					
A. Policy & governance	Indicator A1			Indicator A2		
	Political commitment OAT is included in national drug control, HIV and/or hepatitis strategies and action plans, with a commitment to WHO-recommended targets Legislation explicitly supports the provision of OAT OAT is a core part of national policy for opioid dependence management Law enforcement and justice systems support implementation and expansion, as needed, of OAT Effective governance and coordination oversee the development of OAT in the country Civil society, including OAT clients, are consulted in OAT governance and coordination at country level			Management of transition from donor to domestic systems Country has adopted a plan which defines transition of OAT from donor to domestic funding including a timeline There is a multi-year financial plan for the OAT transition to domestic sources, with unit costs developed, co-financing level, the (future) domestic funding sources for OAT identified and agreed among country representatives Donor transition oversight in the country effectively supports implementation of the OAT transition to domestic systems There is good progress in the implementation of the OAT-component in the transition plan		
B. Finance & resources	Indicator B1: Medications	Indicator B2: Financial resources		Indicator B3: Human resources		Indicator B4: Evidence and information systems
	OAT medicine procurement is integrated into domestic PSM system and benefits from good capacity without interruptions Both methadone and buprenorphine are registered and their quality assurance system is operational Methadone and buprenorphine are secured at affordable prices	Methadone and buprenorphine are included in the state reimbursed medicine lists and are funded from public sources OAT services are included in universal health coverage or state guaranteed package of healthcare including for people without health insurance OAT services are paid through sustainable public funding sources which secure adequate funds to cover comprehensive services In the countries with active HIV grants, OAT services are co-financed by the Government in accordance with the Global Fund Sustainability, Transition and Co-Financing Policy		OAT is included in the job description of main health staff and core functions of the state system for drug dependencies with relevant capacities to prescribe and dispense OAT to a required scale Capacity building system is adequate for OAT implementation in a sustainable way		OAT monitoring system is in place and is used for managing the OAT program including program need, coverage and quality assurance Evidence-base for OAT effectiveness and efficiency are regularly generated and inform policy and program planning OAT client data are stored in a database; they are confidential, protected and not shared outside of the health system without a client’s consent
C. Services	Indicator C1: Availability and coverage		Indicator C2: Accessibility		Indicator C3: Quality and integration	
	OAT is available in hospitals and primary care; take-home doses are allowed Coverage of estimated number of opioid dependent people with OAT is high (in line with WHO guidance: 40% or above) OAT is available in closed settings (including for initiation onto OAT), during pre-trial detention and for females OAT is possible and available in the private and/or NGO sectors in addition to the state sector		There are no people on a waiting list for entering the service Opening hours and days accommodate key needs Geographic coverage is adequate There are no user fees and barriers for people without insurance OAT is available and, in general, accessible for populations with special needs (pregnant and other women, sex workers, underage users, ethnic groups) Illicit drug consumption is tolerated (after dose induction phase) Individual plans are produced and offered, with involvement of the service user OAT inclusion criteria are supportive of groups with special needs and are not restrictive, i.e., failure in other treatment programs is not required prior to enrolling into the OAT program		Adequate dosages of methadone/buprenorphine are foreseen in national guidelines and practice in line with WHO guidance OAT programs are based on the maintenance approach and have a high retention of users A high proportion of OAT maintenance sites is integrated and/or cooperates with other services and support continuity of care for HIV, tuberculosis and drug dependency (in line with WHO guidance: 80% or more of the sites) A high proportion of OAT clients receive psycho- and social support (in line with WHO guidance: 80% or more of the sites)	

2.

## Abbreviations

AIDS: Acquired immunodeficiency syndrome
EECA: Eastern Europe and Central Asia
EHRA: Eurasian harm reduction association
GDP: Gross domestic product
Global Fund: Global Fund to Fight HIV/AIDS, Tuberculosis and Malaria
HIV: Human immunodeficiency virus
NGO: Non-governmental organization
OAT: Opioid agonist therapy
TB: Tuberculosis
UHC: Universal health coverage
UNDP: United Nations Development Programme
WHO: World Health Organization

## Footnotes

1 The terms opioid substitution therapy and medically assisted therapy are also sometimes used for this treatment in scientific literature, policy documents and among practitioners. Due to stigma and politicization attached to the former term and the inherent lack of distinction from other approaches when using the second term, this article uses the term opioid agonist therapy.

2 All UN member states in Central and Eastern Europe and Central Asia have ratified the UN Covenant on Economic, Social and Cultural Rights.
