# Correction: Measuring sustainability of opioid agonist therapy programs in the context of transition from Global Fund Support

**DOI:** 10.1186/s12954-024-00945-8

**Published:** 2024-01-31

**Authors:** Raminta Stuikyte, Ivan Varentsov, Catherine Cook, Sergii Dvoriak

**Affiliations:** 1 Vilnius, Lithuania; 2 Eurasian Harm Reduction Association (EHRA), Vilnius, Lithuania; 3 Harm Reduction International, London, UK; 4 Ukrainian Institute On Public Health Policy, Kiev, Ukraine

**Correction: Harm Reduction Journal (2024) 21:7** 10.1186/s12954-024-00931-0

Following publication of the original article [1], the title was incorrectly given as “Measuring sustainability of opioid agonist therapy programs in the context of transition from global fund support” but should have been “Measuring sustainability of opioid agonist therapy programs in the context of transition from Global Fund support”.

The original article has been corrected.
